# Is it possible to make unconscious inferences? The limits of the influence of unconsciously perceived stimuli on conscious decisions

**DOI:** 10.1093/nc/niag068

**Published:** 2026-10-26

**Authors:** Michal Remiszewski, Jan Łacny, Franciszek Cwalina, Kinga Masłowska, Axel Cleeremans, Marek Binder

**Affiliations:** Consciousness Lab, Consciousness Lab, Institute of Psychology, Jagiellonian University, 6 Ingardena Street, 30-060 Kraków, Poland; Institute of Philosophy, Jagiellonian University, 52 Grodzka Street, 31-044 Kraków, Poland; Institute of Philosophy, Jagiellonian University, 52 Grodzka Street, 31-044 Kraków, Poland; Consciousness Lab, Consciousness Lab, Institute of Psychology, Jagiellonian University, 6 Ingardena Street, 30-060 Kraków, Poland; Consciousness, Cognition and Computation Group, Center for Research in Cognition & Neuroscience, Faculty of Psychology and Education, Université Libre de Bruxelles, 50 Avenue Franklin D. Roosevelt, CP 191, 1050 Brussels, Belgium; Consciousness Lab, Consciousness Lab, Institute of Psychology, Jagiellonian University, 6 Ingardena Street, 30-060 Kraków, Poland

**Keywords:** unconscious perception, decision-making, decision complexity, masking, functional role of consciousness

## Abstract

Stimuli presented outside conscious awareness can influence behavior, yet the extent to which such information supports complex decision-making remains unclear. The present study examined the limits of unconscious influence across three levels of decision complexity. Participants made decisions based on stimuli presented using sandwich masking. Stimulus presentation parameters were held constant across conditions, while decision requirements varied. Simple decisions required no contextual integration, intermediate decisions required the integration of masked information with consciously presented context, and complex decisions additionally required logical inference. The results showed that decision accuracy decreased with increasing complexity: it was highest in the simple condition (58%, with strong Bayesian evidence for above-chance performance), lower but still above chance in the medium condition (54%), and not reliably above chance in the complex condition. This suggests that unconscious influence can extend beyond simple decisions but becomes increasingly unreliable when decisions require more demanding integration or inferential processing. Our findings indicate that one of the functional roles of consciousness may be to support the flexible use of information in higher-order cognitive processes and are broadly consistent with theoretical approaches such as the Global Neuronal Workspace Theory.

**Significance Statement**

**Advancing the discipline:** The study advances our understanding of consciousness by testing how far unconsciously perceived information can be used in decision-making. Identifying this potential boundary is important for understanding the nature of unconscious perception and the function of consciousness.

**Novelty:** The novel aspect is that the experiment compares various levels of decision complexity without changing the settings of subliminal stimuli presentation. Previous analyses of the limits of unconscious perception were based on comparisons between different paradigms, which made conclusions more difficult to formulate.

**Importance:** The study may influence discussions about the function of consciousness and may also provide support for theories of consciousness. It is relevant to researchers working on unconscious perception, evolution of consciousness, and theories of consciousness.

**Highlights**

- Unconsciously presented premises influenced participants’ decisions.
- Three levels of decision complexity were tested: simple decisions without context, medium decisions with context, and complex decisions requiring inference.
- Decision accuracy declined as task complexity increased.
- Above-chance performance occurred only in the simple (58%) and medium (54%) conditions, while complex inference-based decisions were not reliably above chance.
- Information integration and logical inference may mark the limits of using unconsciously perceived stimuli.

## Introduction

Can perception occur unconsciously? This question might seem contradictory because we intuitively associate perception with conscious experience, often treating the two as synonymous. However, substantial medical and experimental evidence suggests that consciousness is not necessary for perception. Such unconscious perception involves the processing of stimuli that do not reach conscious awareness but nonetheless influence behavior, reaction times, decision-making, or physiological responses. In this study, we explored to what extent unconsciously perceived information can be used in decision-making. In other words, we investigated how complex decisions can be when made using unconsciously perceived stimuli.

### Unconscious perception

The most spectacular demonstration of unconscious perception is probably the phenomenon of blindsight. Blindsight patients, who suffer from extensive lesions in striate cortex (Marshall and Halligan 1988, Overgaard et al. 2008, Ajina and Bridge 2017), often report that they have lost visual phenomenology. Nevertheless, they can interact with their environment to some extent. For instance, the well-known patient TN was able to fluently avoid obstacles in a corridor (de Gelder et al. 2008).

Unconscious perception is a widely studied topic in experimental psychology. Various methods have been developed to render stimuli subliminal, including masking (see, e.g. Marcel 1983), continuous flash suppression (Tsuchiya and Koch 2005), or tachistoscopic presentation (Beauny et al. 2020). One of the phenomena that has attracted the most attention is subliminal priming, where an unconsciously presented prime influences reaction times to subsequently presented target words (Kiefer 2002). Similar effects with numbers as targets were demonstrated by Dehaene et al. (1998). In their experiment, subliminally presented numbers facilitated participants’ ability to determine whether a target number was higher or lower than 5. The results of these experiments suggest that we can process words and numbers at a relatively high cognitive level even if we do not consciously perceive them (however, subsequent work questioned whether such effects reflect flexible semantic processing, proposing instead that they demonstrate a more constrained form of unconscious perception based on the automatic activation of prespecified stimulus–response mappings; Kunde et al. 2003, Van Opstal et al. 2005).

A wide range of other effects has been reported. For example, unconscious priming has been demonstrated to occur when participants react to colorful figures. When the figures were preceded by other, presumably invisible figures with the same color, the reaction times of the participants were faster than when the colors were different (Ro et al. 2009). Some experiments have focused on the integration of new information and decision-making. In the study by Ruch et al. (2016), the researchers asked participants to estimate the income of people whose faces were displayed on the screen. Before this, the same faces were presented subliminally with a profession name displayed below them, such as a manager or a postman. The results showed that the name of the profession, which was not consciously perceived by the participants, influenced their later estimation of income. In other words, the integration of the unconsciously perceived picture and word meaning allowed participants to make more accurate decisions.

Unconscious perception has been reported in many more studies, including the subliminal presentation of pictures, words, and sounds (see, e.g. Deacon et al. 2000, Naccache and Dehaene 2001, Henke et al. 2003, Chartrand et al. 2006, Karremans et al. 2006, Gibbons 2009, Hughes et al. 2009, Kiefer et al. 2009, Lui and Rosenfeld 2009, Duss et al. 2011, Reber et al. 2014, Ortells et al. 2016, Barutchu et al. 2018, Kawakami et al. 2018, Scott et al. 2018, Wuethrich et al. 2018).

### Limits of unconscious perception

One of the open questions in the field concerns the limits of the influence of unconsciously perceived information on our behavior. A promising strategy to address this issue is to review existing experiments on unconscious perception, classify them by task complexity and difficulty, and identify the level of task difficulty at which the effects of unconscious perception diminish. However, different experiments employ different methodologies (e.g. different methods for rendering stimuli subliminal), which complicates drawing reliable conclusions from such comparisons. In fact, in many experiments, effects of unconscious perception have not been found (see, e.g. Haase and Fisk 2015, Gelbard-Sagiv et al. 2016, Sand 2016), but it is unknown why. It is possible that the studies in which no effects were found genuinely reflect the limits of processing unconsciously perceived stimuli; however, other potential explanations also exist. For instance, the lack of effects may arise from overly strict methods of rendering stimuli subliminal or from overly distracting experimental procedures.^1^ Reliable assessment of the limits of unconscious perception requires using consistent methods of stimulus presentation while manipulating only the complexity of the task.

Although we do not yet know the limits of the influence of unconsciously perceived stimuli on our behavior, the question is crucial for consciousness research. For instance, we often assume that the primary function of consciousness is to interact with the external world (Tye 1999). When we stop at a crossing, we do so because we perceive a red light. If unconscious perception truly occurs—allowing us to perceive colors without conscious awareness or integrate subliminally presented information—this suggests that the role of consciousness may be specific to certain types of perception or particular cognitive processes, such as those involving higher-order reasoning.

What is more, assessing the limits of unconscious perception’s influence may provide valuable evidence in debates between theories of consciousness. For example, the Global Neuronal Workspace Theory (GNWT) posits that global broadcasting of information across distributed brain networks is a necessary condition for consciousness (Dehaene and Naccache 2001, Mashour et al. 2020). If unconscious perception can support only simple processing but not the complex integration or reasoning that require this global broadcasting, it would support GNWT’s claim that consciousness is necessary for higher-order cognitive functions. On the other hand, if unconscious processes can perform tasks previously thought to require global broadcasting, this might challenge GNWT and lend support to alternative theories.

### Current study

The goal of the study described in this paper was to determine how complex actions, specifically decisions, can be influenced by unconsciously perceived stimuli. In other words, we were interested in the limits of the influence of unconsciously perceived stimuli on behavior. To explore this issue, we designed an experiment in which participants made decisions about products, services, or social situations. The experiment included three conditions where the same stimuli presentation methods were used, but the complexity of the decisions varied. Thus, the study compared the effects of unconscious perception on various levels of decision complexity without changing the methodology and settings of the stimuli presentation.

Our research question focused on identifying the level of decision complexity at which the influence of subliminal stimuli remains significant and the point at which it disappears. We hypothesized that the effects of unconscious perception would decrease as decision complexity increases. Specifically, we expected that these effects would disappear in the most complex condition, where unconsciously perceived information needed to be used in reasoning (see the Experimental Procedure section for the description of all the conditions). Since our study manipulated the complexity of decisions without manipulating the parameters of stimulus presentation, it may offer new insight and fill unexplored areas in unconscious perception research.

## Methods and materials

### Participants and equipment

We tested 40 native Polish speakers (29 women, 11 men, mean age = 24 years, SD = 2.7) recruited through the internal recruitment system of Jagiellonian University and through websites with job advertisements. Participants were paid 50 PLN, which is a typical reimbursement in Poland for a study of this duration. The participants were randomly assigned to one of the eight versions of the procedure, which was necessary to counterbalance all the versions of the stimuli (see the Stimuli section). None of the participants was excluded from the study. The experiment was conducted in accordance with the principles of the Declaration of Helsinki. The research protocol was reviewed and approved by the Jagiellonian University research ethics committee.

The procedures were displayed on a Dell Alienware 25″ liquid crystal display (LCD) monitor with a refresh rate of 240 Hz, which allows presenting stimuli with a temporal resolution of 4.17 ms. The timing accuracy of the monitor display was tested before the experiment with the use of a photosensitive diode. The test confirmed that the desired stimulus durations are controlled with sufficient temporal precision. The distance between the participant face and the screen was kept at ~80 cm, resulting in presentation of stimuli at a visual angle between 4° (single words) and 15° (other elements of the procedure, see the Experimental Procedure section). All the stimuli were displayed using white and red font colors on a black background. Participants responded to the stimuli using a mouse.

### Experimental procedure

The main part of the experiment, referred to as the Decision Task, lasted 50 min (80 min including the calibration and the objective test of awareness; see the Awareness Measures section), during which participants were presented with both supraliminal and subliminal stimuli. They were instructed to read all the information displayed on the screen and to make decisions by answering the questions that appeared at the end of each trial. Participants interacted with the task using a mouse. The task began with a brief training session, during which stimuli were presented alongside specific instructions and comments that informed participants about the exact structure of trials.

There were three conditions representing different levels of decision complexity: (i) SIMPLE: decisions without context, (ii) MEDIUM: decisions with context, and (iii) COMPLEX: decisions with context that required inference. Each condition consisted of 20 decisions, for a total of 60 decisions. The decisions from each condition were randomly mixed.

#### Simple condition

At the beginning of each trial in the simple condition, participants were presented with the Main Sentence Preview (see Fig. 1), which informed them about what they would soon read, for example: “In a moment, you will learn that Edward is stupid or that Edward is clever.” At this point, participants had no information about the subject of the sentence—Edward. After they pressed the mouse button, a second screen appeared, which we refer to as the Question Preview. The Question Preview was displayed to facilitate the subsequent decision (see below for more details). For example, the following information might have been presented during the Question Preview: “In this trial, you will be asked the following question: Would you advise your friend to hire Edward?” Participants were also shown reminders repeating the information from the Main Sentence Preview (see Fig. 1: First Reminder and Second Reminder).

**Figure 1:**
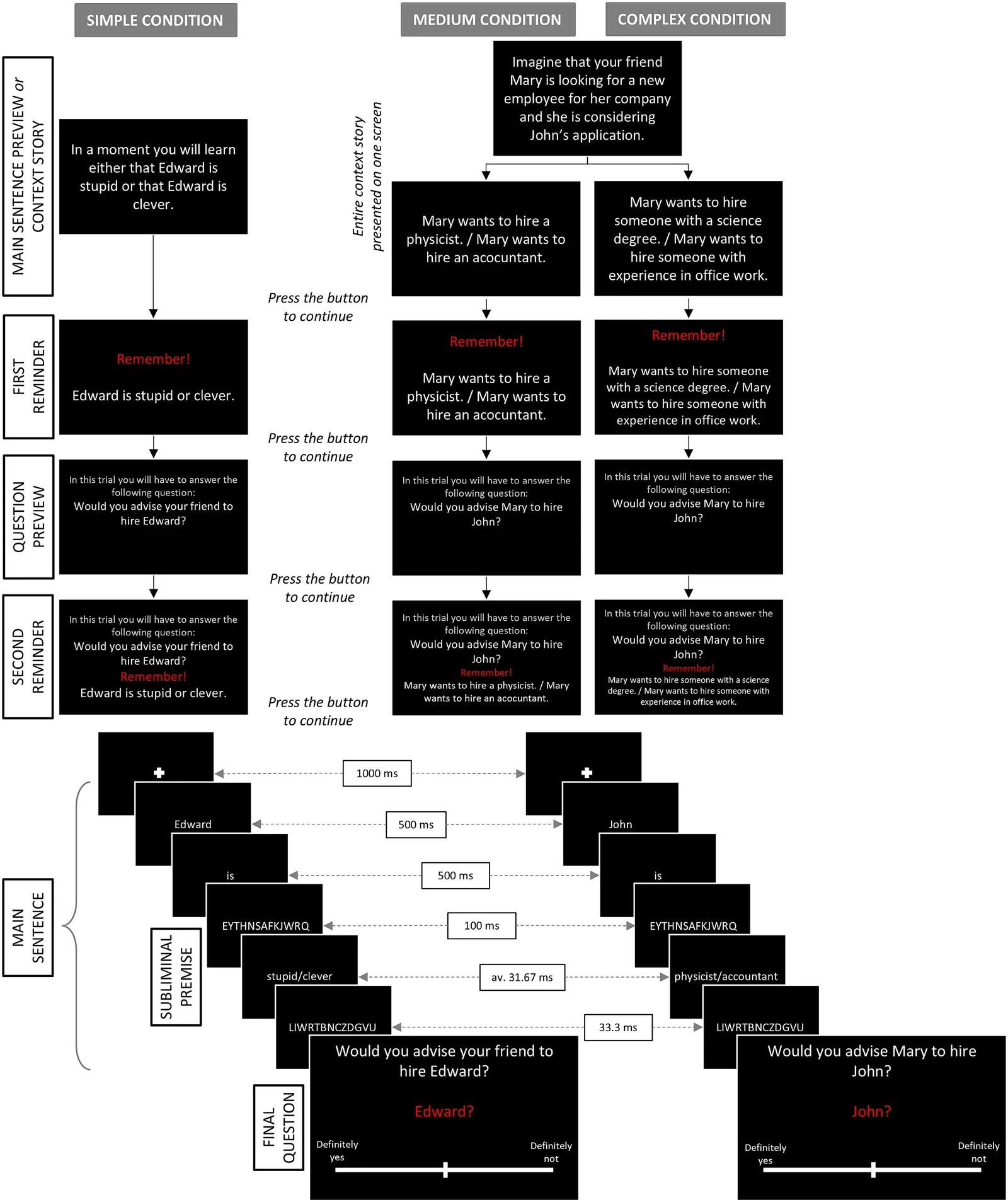
The three conditions of the Decision Task are indicated by labels at the top of the graph, while the elements of the stimuli (Main Sentence Preview/Context Story with supraliminal premises, First Reminder, Question Preview, Second Reminder, Main Sentence, Subliminal Premise, and Final Question) are represented by vertical labels. The smaller labels in the center show the presentation times for each screen. The remaining screens stay displayed until the participant presses a mouse button. In the simple condition, the Context Story is replaced with the Main Sentence Preview. The medium and complex conditions share the same Context Story but differ in the supraliminal premise (for example, “Mary wants to hire a physicist” in the medium condition). Although the graph suggests that the supraliminal premises are presented on the next screen, they are in fact presented on the same screen as the beginning of the Context Story (for example, “Imagine that your friend Mary is looking for a new employee for her company and she is considering John’s application. John is a physicist.”). Note that the subliminal words in the Main Sentences do not contain articles—the task was conducted in Polish, in which articles do not occur. The final question screen contains the slider, the question, and the shortened question written in red font to increase the probability of semantic bonding and increase masking effectiveness.

Then, the Main Sentence appeared sequentially, word by word: “Edward is X,” where X was replaced with the Subliminal Prime, either “stupid” or “clever.” The subliminal word was masked using a sandwich masking technique. Both masks consisted of random strings of capital letters. Immediately after the second mask disappeared, a decision screen was displayed with the Final Question: “Would you advise your friend to hire Edward?” The only information needed for a correct answer was the ending of the Main Sentence—the Subliminal Premise. “Stupid” is clearly a negative word, which would imply that the correct answer is NO, while “clever” is a positive word, indicating that the answer should be YES.

We aimed for participants to respond quickly, as slower reactions could diminish the effects of unconscious perception (Kiefer 2002, Meade and Coch 2017). This is why we presented the Question Preview at the beginning of each trial. The Question Preview allowed participants to familiarize themselves with the question, enabling them to answer more quickly when it was displayed on the screen.

In all conditions, participants used a slider to provide their answers (see Fig. 1). The left end of the slider was labeled “DEFINITELY YES,” while the right end was labeled “DEFINITELY NO.” The slider was used solely to increase participants’ comfort when responding. It allowed them to click near the center—on either side—which could be more natural when they were uncertain about their choices (it was not possible to click on the exact center of the slider). This flexibility was important, as participants were not consciously aware of the sentence endings and, during the pilot studies, some subjects reported that they felt as the task does not make sense at all. Thus, if the participants were forced to give simple YES/NO answers, it could potentially have caused frustration and negatively impact the performance during the task.

#### Medium condition

At the beginning of each trial in the medium condition, unlike in the simple condition, there was no Main Sentence Preview. Instead, participants were presented with a Context Story, such as: “Imagine that your friend Mary is looking for a new employee for her company and is considering John’s application. Mary wants her new employee to be X,” where X was replaced with either “a physicist” or “an accountant.” We refer to X as the Supraliminal Premise. Then, similarly to the simple condition, participants were presented with the Question Preview to inform them of the decision they would need to make at the end of the trial. For example, the following information might be displayed: “In this trial, you will be asked the following question: Would you advise your friend to hire John?” The First Reminder and the Second Reminder were also presented between these screens (see: Fig. 1).

When participants pressed the mouse button after reading the context information, the Main Sentence was displayed sequentially, word by word: “John is Y,” where Y was replaced with the Subliminal Premise, either “a physicist” or “an accountant” (it is worth noting that articles—such as “a,” “an,” and “the”—do not occur in Polish). Immediately after the presentation of the Subliminal Premise, the decision screen appeared with the Final Question: “Would you advise Mary to hire John?” The correct answer was YES when X (the Supraliminal Premise) and Y (the Subliminal Premise) were the same, and NO when they were different. For example, if Mary wants her new employee to be a physicist and John is a physicist, we can advise Mary to hire John.

The medium condition is more complex than the simple condition because the Context Story is necessary for making a correct decision. For instance, being an accountant typically carries no inherent positive or negative connotations, so additional information is needed to solve the task accurately. In other words, the correct answer requires the integration of the Context Story—especially the Supraliminal Premise—with the Subliminal Premise. In the simple condition described previously, context was not necessary to answer the question, which is why there was no Context Story and only the Main Sentence Preview. The only information that mattered in the simple condition was the Subliminal Prime at the end of the Main Sentence.

#### Complex condition

The complex condition—decisions with context that required inference—followed the same pattern as the medium condition, but the Context Story contained a less specific Supraliminal Premise, for example: “Imagine that your friend Mary is looking for a new employee for her company. Mary wants to hire someone with X.” In this case, X (the Supraliminal Premise) could be either “a science degree” or “experience in office work.” The Main Sentence remained the same as in the medium condition: “John is Y,” where Y (the Subliminal Premise) could be either “a physicist” or “an accountant.” Following the presentation of the Main Sentence, the Final Question appeared on the screen.

The key difference between the medium condition and the complex condition lies in the Supraliminal Premises. “Someone with a science degree” (one of the Supraliminal Premises occurring at the end of the Context Story) is a category that may include a physicist, as well as a chemist or a biologist. Similarly, “someone with experience in office work” (the alternative Supraliminal Premise) is also a category that contains elements such as an accountant, a clerk, or a bookkeeper. Therefore, the Context Story in the complex condition does not explicitly indicate the correct answer, unlike in the medium condition, where precise indications appear (e.g. “Mary wants to hire a physicist”). Here, participants must infer the correct answer from the provided information, which makes this condition the most complex of the three. Specifically, they need to determine whether the subliminal word (the Subliminal Premise) is a valid instance of the category defined by the Context Story—not just whether the Subliminal Premise matches the Supraliminal Premise. The correct answer can only be reached through logical inference, for example: *John is a physicist. → Mary wants to hire someone with a science degree. → Physicists have science degrees. → John meets the Mary’s requirements.*

#### Nuances of the design

To summarize, in the medium condition, the Supraliminal Premise indicates the exact preference (“*Mary wants to hire someone who is a physicist.*”), while in the complex condition it indicates a general preference (*“Mary wants to hire someone with a science degree.”*). In the simple condition, in turn, there is no Supraliminal Premise and no Context Story, which is replaced by the Main Sentence Preview. The Main Sentence Preview fills the gap between the Question Preview and the Main Sentence in the simple condition. It also gives participants a clue about what the Main Sentence might be (the Supraliminal Premise is such a clue in the medium and the complex condition).

In all conditions, participants were presented with reminders throughout the trial. For example, in the “Mary and physicist” trial, after the Context Story, an additional screen appeared with the message: “Remember! Mary wants to hire a physicist.” Following this, participants saw a reminder of the upcoming question and a second reminder about the Context Story. We included reminders in the trials because we realized that some participants tend to read very quickly, and we wanted to minimize situations in which they might forget the key information necessary to make a quick decision at the end of a trial.

During the procedure, there were 15 catch trials (randomly presented among regular trials) in which participants were shown a final word of a Main Sentence supraliminally (instead of masks, a final word was displayed). The purpose of these catch trials was to assess whether participants were paying attention to the stimuli and carefully reading all the information displayed on the screen.

### Stimuli

The subliminal character of the premises was achieved by a sandwich masking technique. The Subliminal Premises were masked by random strings of capital letters (e.g. PBAFNFAMNKWJQ); the pre-mask duration was 100 ms, while the post-mask duration was 33.3 ms (see: Kiefer 2002). After the post-mask, the decision screen appeared with the Final Question in the upper part of the screen. Simultaneously, a shortened version of the question, written in red font, was displayed at the center of the screen, exactly where the post-mask had previously been shown (this element is not shown in Fig. 1). The red, shortened question served several purposes. It enhanced the masking effect of the post-mask (as in the Kiefer 2002 study and many other experiments in which targets are displayed immediately after subliminal stimuli), and it increased the likelihood that the Subliminal Premise (e.g. “physicist”) would be associated with the subject of the question (e.g. “John”), since both words (the Subliminal Premise and the subject of the question) appeared close in time. Additionally, the shortened question allowed participants to respond more quickly, as they were already familiar with the full version of the question from the Question Preview at the beginning of the trial. 

**Table 1:** Four stimulus versions in the simple condition and eight stimulus versions in the medium and complex conditions (numbers in brackets indicate the version of a stimulus)

**Condition**	**The Main Sentence Preview/the Context Story**	**The Supraliminal Premise**	**The Main Sentence**	**The Subliminal Premise**
SIMPLE: no context	In a moment you will learn either that Edward is. . .	. . .clever or that Edward is stupid.	Edward is. . .	. . .stupid. (1)
				. . .clever. (2)
		. . .stupid or that Edward is clever.		. . .stupid. (3)
				. . .clever. (4)
MEDIUM: context	Mary wants to hire someone who is. . .	. . .a physicist.	John is. . .	. . .physicist. (1)
				. . .accountant. (2)
		. . .an accountant.		. . .physicist. (3)
				. . .accountant. (4)
COMPLEX: context + inference	Mary wants to hire someone with. . .	. . .a science degree.		. . .physicist. (5)
				. . .accountant. (6)
		. . .an experience in office work.		. . .physicist. (7)
				. . .accountant. (8)

The duration of the Subliminal Premise for each participant was calibrated using the calibration protocol (see the Awareness Measures section), resulting in an average subliminal stimulus duration of 31.67 ms (SD = 6.17 ms).

There were four versions of each stimulus in the simple condition and four versions of each stimulus in the medium and complex conditions (see Table 1). Moreover, each stimulus of the medium condition had its counterpart in the complex condition (and *vice versa*). For example, the Context Story about Mary deliberating whether to hire John appears in both the medium and complex conditions. The reason for this is that we wanted to manipulate the complexity of the decisions, not the content of the stimuli. Due to practical reasons, it was not possible to extend this rule to the simple condition as well. As a result, there were four combinations of stimuli in the simple condition and eight combinations of stimuli in the medium and complex conditions. All combinations of stimuli are listed in Table 1.

The stimuli were counterbalanced between the participants. It was never the case that the stimulus about the same protagonist (e.g. Mary who wants to hire someone) was in both, medium and complex conditions in the same participant—only one version of a stimulus appeared during a single experimental session.

The stimuli were designed with the aim of simulating decisions people face in everyday life, as far as this is achievable in a laboratory setting. This objective shaped the overall structure of the task. To minimize interference between the Subliminal Premises and participants’ genuine preferences, we avoided asking direct questions (e.g. “Would you hire this person?”). Instead, participants were asked to give advice to hypothetical individuals. English names were used—even in the Polish version of the task—to reduce the likelihood that any presented names would match those of participants’ real-life friends.

To summarize, in order to make correct decisions, participants had to use subliminally presented stimuli (the Subliminal Premises). In the simple condition (decisions without context), the Subliminal Premise did not need to be integrated with any context. The condition was designed in such a way that unconscious processing of the meaning of the Subliminal Premise was sufficient to correctly answer the Final Question. In the medium condition (decisions with context), in turn, an unconscious representation of the Subliminal Premise had to be integrated with information from the Supraliminal Premise, stored within the working memory during the course of a trial. The medium condition was more complex than the simple condition, as the adequate decisions were not based solely on the meanings of the Subliminal Premises. Finally, to answer a question in the complex condition (decisions with context that require inference)*,* the Subliminal Premise had to be a part of a simple logical inference, making this condition the most complex of the three. Therefore, in each condition, the Subliminal Premises had to be processed at different cognitive levels.

### Awareness measures

#### Objective test of awareness

Subjective tests of awareness typically involve either asking participants whether they perceived the subliminal stimulus or having them rate their awareness using a scale such as the Perceptual Awareness Scale (Ramsøy and Overgaard 2004). The standard argument against subjective measures is the criterion problem, according to which different participants may use different criteria to report the visibility of stimuli (Björkman et al. 1993, Kunimoto et al. 2001, Hannula et al. 2005, Peters and Lau 2015, Timmermans and Cleeremans 2015, Balsdon and Clifford 2018). For example, some people may claim that they failed to see a stimulus whereas in fact, they had a degraded conscious perception of something that resembled a stimulus. A solution to the criterion problem is to use objective measures of awareness, through which participants are forced to decide which of the two (or more) possible stimuli (or categories of stimuli) was presented subliminally. For example, participants may be asked whether a subliminal stimulus was a word or a pseudoword (Kiefer et al. 2009) or a random string of letters (Naccache and Dehaene 2001).

In this study, we used a variation of the Lexical Decision Task (LDT) as an objective measure of awareness of subliminal stimuli, following previous research that employed the LDT for this purpose (Dehaene et al. 1998, Kiefer 2002). The LDT allows assessment of whether participants can consciously discriminate the stimuli as words versus pseudowords. Failure to reliably distinguish between these categories indicates lack of conscious access to the lexical information conveyed by the stimulus and thus to information necessary for semantic processing.

This rationale is particularly relevant in the context of the present paradigm. Performance in the Decision Task depends on the semantic relation between the target word and the question context, which constitutes the critical stimulus feature driving the effect. Conscious processing of this relation presupposes that the stimulus is identified as a word. The LDT therefore tests a precondition for conscious access to the meaning relevant for task performance. This approach is consistent with recent methodological recommendations emphasizing that awareness measures should target the critical stimulus feature underlying the indirect effect (Schmidt and Biafora 2024).

The LDT simulated elements of the Decision Task to align the test conditions with those of the main experiment. Participants read sentences presented word-by-word, with the last word masked, exactly as in the Decision Task. Instead of the shortened question in red font, a red prompt encouraging a response (e.g. ‘make a choice’) was displayed (not shown in Fig. 2). In half of the trials, the masked stimulus was a word, and in the other half, it was a pseudoword. For instance, participants might see the sentence “Claire eats X,” where X was either the word “lettuce” or the pseudoword “wradon.” The words used in the LDT were not the same as in the main experiment; however, they were matched to the experimental words in terms of the number of syllables. Similarly, the pseudowords always matched the words in syllable count. Participants used the left and right shift buttons on a keyboard to indicate whether the masked stimulus was a word or a pseudoword.

**Figure 2:**
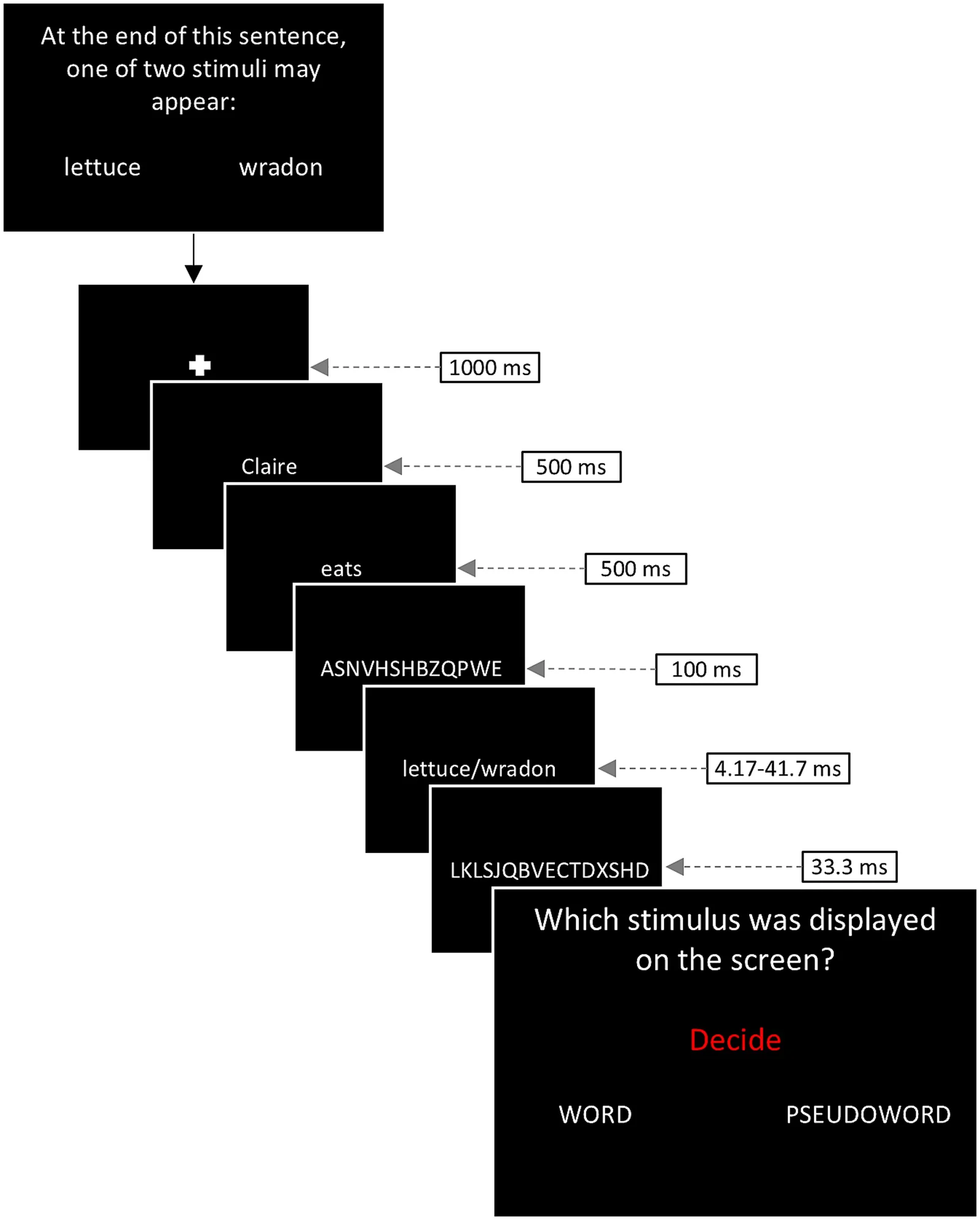
The Lexical Decision Task served as the objective test of awareness. Each trial began with the presentation of two possible sentence endings. After participants read this information, they had to press a space bar on a keyboard to initiate the word-by-word presentation of the sentence. At the end of each trial, participants used the shift buttons to decide what was displayed at the end of the sentence. The subliminal word presentation time was determined during the calibration protocol. The word in the middle of the final screen (displayed in red font) was intended to simulate the shortened question used in the indirect task, thereby increasing the effectiveness of masking. The specific word varied across trials; several variants were used (e.g. decide, choose, select, respond).

At the beginning of the task, participants were informed that words would appear in half of the trials. Before each sentence was presented, they were also told which specific word or pseudoword might appear at its end. This setup mirrors the structure of the Decision Task, in which each trial begins with a short contextual story that suggests a plausible sentence ending (e.g. “Mary wants to hire a physicist” implies that the ending might be “a physicist” or an alternative). The LDT consisted of 53 or 60 trials, a number close to that used in the Decision Task.^2^

#### Calibration of subliminal stimuli duration

Each experimental session began with calibrating the duration of stimuli to reduce the probability that participants would see subliminal words. During calibration, participants repeatedly performed the LDT (described above) to determine the duration at which the masked stimulus remained invisible. Initially, in the first run, the presentation time for subliminal words/pseudowords was set to 33.3 ms. After 60 trials (the same number as in the Decision Task), the procedure calculated accuracy (percentage of correct answers). If accuracy exceeded 55%, the duration of the subliminal stimulus was shortened by 4.17 ms (1 frame on a 240 Hz monitor), and the test was repeated. If the participant’s accuracy was below 55%, the stimulus duration remained unchanged, and the test was repeated with the same visibility. If the average score across tests at the same duration dropped below 53%, calibration was completed and the duration for the Decision Task was determined. However, if the average remained above 53%, the duration was again shortened by 4.17 ms, and the test was repeated. From that point on, the algorithm once again followed the “55% rule”: if the score exceeded 55%, the duration was shortened; if it was below 55%, the duration remained unchanged, and the test was repeated at the same duration.

A high score on the awareness test could occasionally have been a statistical artifact. To balance out such occurrences, similar rules were applied to low scores: the “45% rule” for a single run of the test and the “47% rule” for two runs. As a result, if a participant happened to score randomly above 55%, which might have prompted an unnecessary reduction in duration, another participant might have scored randomly below 45%, leading to an unnecessary increase in duration. These effects were expected to offset each other.

The following example illustrates the procedure: one of the participants scored 58% in the first run of the calibration protocol with a subliminal word duration of 33.3 ms. As a result, the presentation time was decreased by 4.17 ms (to 29.13 ms), but the score remained at 58%. The duration was reduced again (to 24.79 ms), resulting in a score of 51%. The test was repeated at the same duration, yielding a score of 56%, which meant that the average exceeded 53%. Consequently, the duration was further shortened to 20.62 ms. The next two scores were 50% and 51%, indicating that the duration of 20.62 ms should be selected for both the Decision Task and the objective test of awareness.

### Data analysis

All statistical analyses were conducted in JASP, version 0.18.3 (JASP Team, 2024). There were two primary outcomes:

1. Accuracy in the Decision Task of each participant in each condition (simple, medium, and complex). The accuracy was calculated in the following way: responses falling on the “correct” side of the slider were marked as correct, while those on the “incorrect” side were marked as incorrect. Participants could not select the central point of the slider. For example, if Mary wanted to hire a physicist and John was a physicist, the correct answer included any point on the left side of the slider (toward the “Definitely yes” response).
2. Accuracy in the objective test of awareness. The accuracy was calculated as the proportion of the correct answers.

The analysis of the Decision Task focused on binarized decision accuracy rather than response magnitude on the slider, as the task itself required a categorical decision (e.g. hire vs. not hire). This approach also reflects methodological considerations discussed in the literature (Meyen et al. 2022), highlighting the importance of using comparable measures in direct and indirect tasks to prevent artefactual dissociations. In both outcomes (the Decision Task and the objective test of awareness), the accuracy was compared to the random response level of 50%. Bayes factors quantifying evidence for a deviation from chance performance were computed in JASP using the two-sided Bayesian *t-*test model with a Cauchy prior on effect size (*r* = 0.7). A weakly informative prior was used, consistent with previous research on unconscious perception, where effects are typically expected to fall within the small-to-moderate range. Following common conventions in the Bayesian literature, values around *BF* ≈ 3 were treated as reflecting at least moderate evidence; however, Bayes factors were interpreted primarily as graded measures of evidence rather than strict decision criteria. The one-sample Bayes factor analyses were complemented by a Bayesian repeated-measures analysis of variance (ANOVA) with CONDITION as a within-subject factor, Bayesian *post hoc* comparisons, and robustness analyses using default and tighter priors.

## Results

### Decision Task

#### Accuracy across decision-complexity conditions

Accuracy in the simple condition (decisions without context) was 57.94% (*SD* = 11.85%), with a Bayes factor of *BF₁₀* = 186.65. This indicates that the observed data were ~187 times more likely under a model including an effect than under the null model, providing very strong evidence that performance exceeded chance in the simple condition. The effect size was large (Cohen’s *d* = 0.67), and the posterior median of the standardized effect size was δ = 0.632, 95% CrI [0.295, 0.976].

Accuracy in the medium condition (decisions with context) was 54.19% (*SD* = 8.74%), with *BF₁₀* = 8.40, indicating moderate evidence that performance exceeded chance. The corresponding effect size was moderate (Cohen’s *d* = 0.48), and the posterior median was δ = 0.448, 95% CrI [0.130, 0.773].

Finally, accuracy in the complex condition (decisions with context that required inference) was 52.31% (*SD* = 10.24%), with *BF₁₀* = 0.44 (*BF₀₁* = 2.30; Cohen’s *d* = 0.23), indicating weak and inconclusive evidence, slightly favoring the null model. The posterior median effect size was smaller, δ = 0.209, and the 95% credible interval included zero, 95% CrI [−0.092, 0.514]. Figure 3 shows the mean accuracy of participants in each condition.

**Figure 3:**
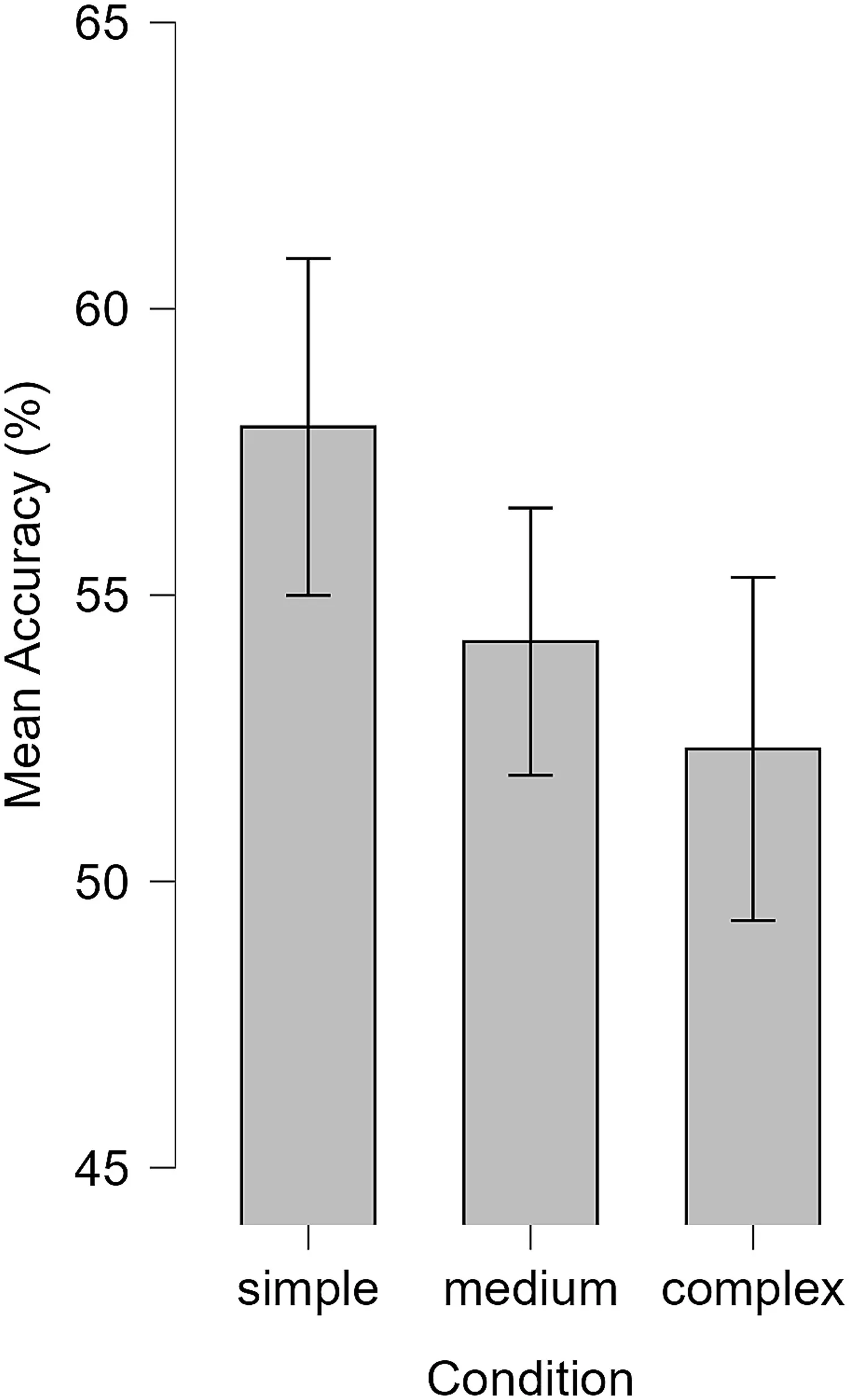
The mean accuracy of decisions in each condition. Chance level is 50%. The left bar indicates the mean accuracy of participants in the simple condition (decisions without context). The middle bar indicates the accuracy in the medium condition (decisions with context). The right bar indicates the accuracy of the decisions in the complex condition (decisions with context that require inference). Error bars represent 95% within-subject confidence intervals.

Sequential Bayesian analyses were conducted to examine how evidence for models with versus without an effect evolved as a function of sample size in each condition (Fig. 4). In the simple condition, the Bayes factor increased steadily with the addition of participants, indicating consistent accumulation of evidence favoring the model including an effect. In the medium condition, evidence initially fluctuated around indifference (BF ≈ 1) and gradually shifted toward support for the model including an effect as sample size increased. In contrast, in the complex condition, the Bayes factor remained close to 1 or favored the null model across most of the sampling process, indicating no systematic accumulation of evidence for an effect.

**Figure 4:**
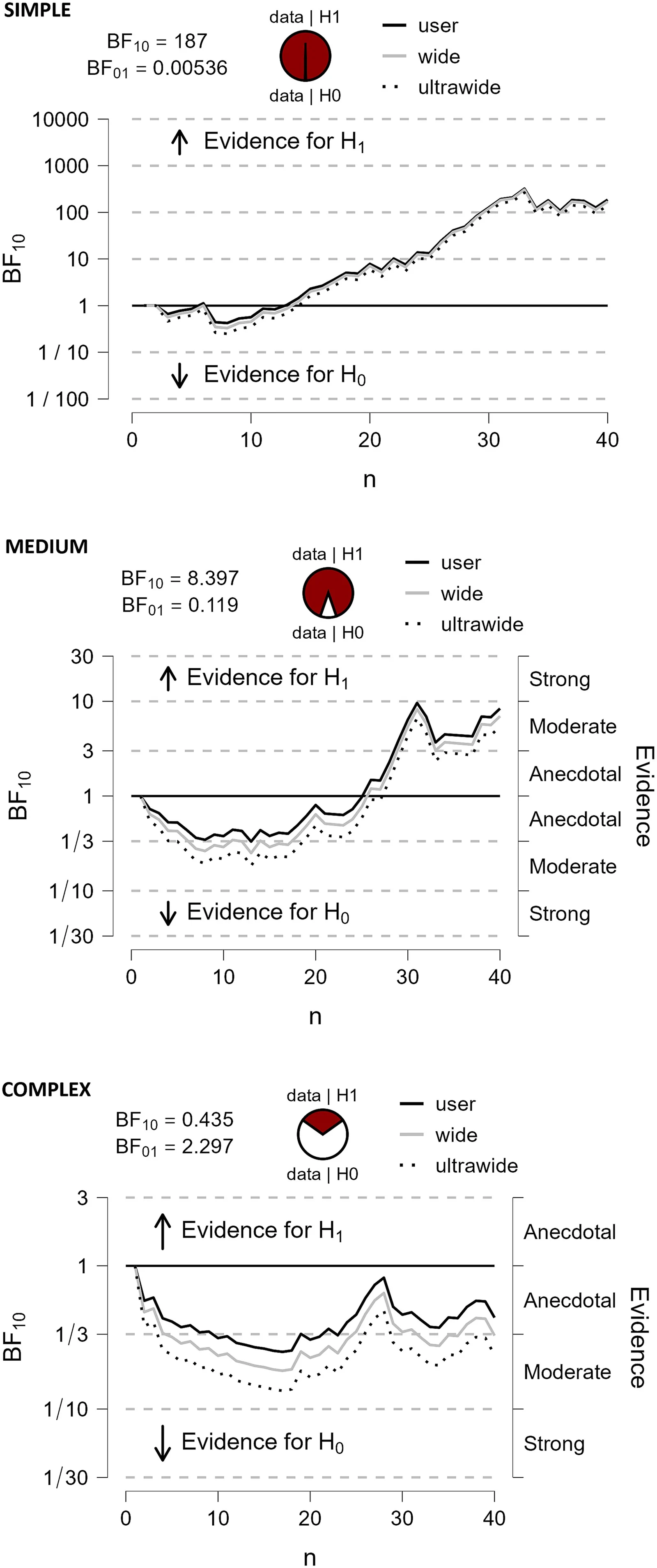
Sequential Bayes factors (BF₁₀) as a function of increasing sample size in the simple (top), medium (middle), and complex (bottom) conditions. Analyses were conducted using a Cauchy prior on effect size (*r* = 0.7), with robustness checks for wider priors (*r* = 1 and *r* = 1.5). The sequential analyses were intended as descriptive evidence-accumulation plots and were not used as a sequential stopping rule.

Additional robustness analyses using wider Cauchy priors yielded the same qualitative pattern (see Fig. 4). With *r* = 1.00 and *r* = 1.50, respectively, evidence for above-chance performance remained very strong in the simple condition, BF₁₀ = 169.57 and BF₁₀ = 135.75. Evidence also continued to favor above-chance performance in the medium condition, although it decreased as the prior became wider, BF₁₀ = 6.97 and BF₁₀ = 5.19. In contrast, the complex condition continued not to provide evidence for above-chance performance, BF₁₀ = 0.33 and BF₁₀ = 0.23, corresponding to BF₀₁ = 3.07 and BF₀₁ = 4.39 for *r* = 1.00 and *r* = 1.50, respectively. Thus, as the prior became wider, the evidence in the complex condition increasingly favored the null model.

We also repeated the one-sample Bayes factor analyses using tighter Cauchy priors on effect size, *r* = 0.50 and *r* = 0.35. The qualitative pattern remained stable across prior specifications. Evidence for above-chance performance remained very strong in the simple condition, BF₁₀ = 185.92 and BF₁₀ = 167.45 for *r* = 0.50 and *r* = 0.35, respectively. Evidence also remained moderate-to-strong in the medium condition, BF₁₀ = 9.34 and BF₁₀ = 9.52, respectively. In contrast, the complex condition did not provide evidence for above-chance performance under either tighter prior, BF₁₀ = 0.56 and BF₁₀ = 0.69, respectively.

Taken together, sensitivity analyses using both tighter and wider Cauchy priors indicate that the main pattern of results was not driven by a specific prior choice. Across prior specifications, evidence for above-chance performance remained strongest in the simple condition, weaker but still present in the medium condition, and unsupported in the complex condition.

#### Relational analysis across complexity conditions

To assess relational evidence for differences between the three decision-complexity conditions, we conducted a Bayesian repeated-measures ANOVA with CONDITION as a within-subject factor. Because Bayes factors can be sensitive to prior specification, we used the default prior for fixed effects, *r* = 0.50, and repeated the analysis using progressively tighter priors, *r* = 0.35 and *r* = 0.15, to assess whether the overall pattern of evidence remained stable across prior assumptions.

Across all three prior specifications, the model including CONDITION was favored over the null model. For *r* = 0.50, the posterior model probability for the model including CONDITION was P(M|data) = .742, BF₁₀ = 2.88. For *r* = 0.35, the corresponding values were P(M|data) = .783, BF₁₀ = 3.62. For *r* = 0.15, they were P(M|data) = .785, BF₁₀ = 3.65. Thus, the Bayesian repeated-measures ANOVA provided consistent evidence for including CONDITION in the model across prior specifications, suggesting that the relational pattern among the three conditions was not dependent on a single prior setting.

Bayesian *post hoc* comparisons were also conducted in JASP. For each comparison, we report the posterior odds provided by JASP together with the corresponding uncorrected Bayes factor, BF₁₀, *U*. The largest pairwise evidence for a difference was observed for the simple–complex comparison, posterior odds = 1.72, BF₁₀, *U* = 2.93. The simple–medium comparison remained inconclusive, posterior odds = 0.74, BF₁₀, *U* = 1.27. The medium–complex comparison favored the absence of a difference, posterior odds = 0.17, BF₁₀, *U* = 0.28. Thus, the Bayesian *post hoc* analyses did not indicate a clear separation between all three levels of decision complexity but rather suggested that the clearest pairwise contrast concerned the least and the most complex conditions.

Because the *post hoc* comparisons did not provide decisive evidence for differences between adjacent levels of complexity, we conducted an additional Bayesian model comparison to examine whether the observed pattern was better characterized as a graded decrease across all three conditions or as a step-like pattern driven primarily by the simple condition. The first model represented a graded/linear decrease across decision complexity, with contrast weights +1/√2, 0, and −1/√2 for the simple, medium, and complex conditions, respectively. The second model represented a step-like/simple-only pattern, in which the simple condition differed from the two more complex conditions, while the medium and complex conditions were treated as equivalent; this model used contrast weights +2/√6, −1/√6, and −1/√6.

Under the default prior, *r* = 0.50, both models were favored over the null model. The graded model yielded BF₁₀ = 7.17 relative to the null model, whereas the step-like model yielded BF₁₀ = 5.26. However, the direct comparison between the two models was inconclusive, BF_graded/step = 1.36. Thus, although the data were compatible with a graded decrease across decision complexity, they did not decisively distinguish this pattern from a step-like account driven primarily by the simple condition.

In summary, the pattern of results, which was stable across different prior specifications, suggests that the influence of masked stimuli was reliably detectable in the simple and medium conditions but not in the complex condition. Although the Bayes factor analysis for the complex condition favored the absence of above-chance performance, the present data do not allow us to conclude that this specific level of complexity constitutes a sharp limit of unconscious processing. This is because the relational analyses did not provide decisive evidence for a difference between adjacent levels of complexity, particularly between the medium and complex conditions. Rather, the results suggest that the boundary of unconscious influence lies beyond the simplest form of decision-making and may emerge when decisions require more complex integration or inferential processing.

#### Complementary analyses

As a complementary frequentist analysis, we also conducted a repeated-measures ANOVA with CONDITION (simple, medium, complex) as a within-subject factor. This analysis revealed a significant main effect of CONDITION, *F*(2, 78) = 4.36, *P* = .016. Follow-up Holm-corrected *post hoc* tests indicated that accuracy was higher in the simple condition than in the complex condition, *P* = .044, whereas the differences between the simple and medium conditions, *P* = .081, and between the medium and complex conditions, *P* = .308, were not statistically significant. To further characterize this pattern, polynomial contrasts were conducted. These revealed a significant linear trend, *t*(39) = −2.55, *P* = .015, indicating that performance decreased as decision complexity increased. The quadratic trend was not significant, *P* = .511. Although this pattern is consistent with a graded decrease across conditions, the Bayesian model comparison reported above did not decisively distinguish the graded model from the step-like model. Therefore, the linear trend should be interpreted cautiously rather than as conclusive evidence for a graded pattern.

As an additional analysis, we examined participants’ continuous responses on the decision slider, which consisted of 20 discrete positions. Responses were coded so that positive values indicated a directional bias toward the correct answer; negative values indicated a directional bias toward the incorrect answer, whereas zero indicated no directional bias. The results closely mirrored those obtained from the binarized accuracy data. One-sample Bayes factor analyses against zero provided very strong evidence for a directional bias toward correct answers in the simple condition, BF₁₀ = 652.80, and strong evidence for such a bias in the medium condition, BF₁₀ = 47.76. In contrast, the complex condition did not provide evidence for a directional bias, BF₁₀ = 0.29, corresponding to BF₀₁ = 3.41.

Finally, we calculated participants’ average accuracy in the catch trials, in which the sentence endings were clearly visible. Accuracy was 93.75% (SD = 14.00%), suggesting that participants carefully read the stimuli presented on the screen.

### Objective test of awareness

We expected that the average accuracy in the objective awareness test would not be significantly different from 50%. The average discrimination score in the objective awareness test was 51.11% (*SD:* 6.31%). The Bayes factor quantifying evidence for a deviation from chance performance (50%) was computed using the Bayesian *t-*test model. The resulting Bayes factor was *BF₁₀* = 0.30 (*BF₀₁* = 3.31), indicating moderate evidence that performance in the awareness test did not differ from chance. This result indicates that the participants were not aware of subliminal stimuli.

Additionally, to ensure sufficient statistical sensitivity at the trial level, we conducted a cumulative chi-square test across participants. The summed chi-square statistic did not differ from chance, *χ*^2^(40) = 35.01, *P* = .69. Converging evidence from both analyses therefore indicates absence of above-chance performance in the objective awareness test.

## Discussion

The purpose of the study was to find the limits of unconscious perception. To do so, we designed a paradigm that allows manipulating the complexity of decisions without changing the parameters of stimuli presentation. There were three conditions: (i) simple: decisions without context, (ii) medium: decisions with context, and (iii) complex: decisions with context that require logical inference.

In the simple condition, participants made decisions based solely on the ending of a sentence (the Subliminal Premise). In the medium condition, the task became more challenging, as participants needed to integrate the Subliminal Premise with the Context Story presented at the beginning of each trial. In the complex condition, participants not only had to merge the Subliminal Premise with the Context Story but also perform simple logical reasoning to answer the question correctly.

Our general hypothesis was that the effects of unconscious perception would diminish as decision complexity increased. In other words, we expected that participants would provide fewer correct answers with the increase of complexity. The results confirmed this hypothesis. With the increase in decision complexity, we observed a decrease in decision accuracy. Accuracy was highest in the simple condition: 58%. This score was significantly higher than chance level (50%). According to Bayesian analysis, evidence for the difference between the random level and the obtained accuracy was extreme. The lower accuracy—although still significantly above the random level—was observed in the medium condition (54%; according to Bayesian analysis, we found substantial evidence for the difference between the obtained accuracy and the random level of the answers). We found no evidence of a difference between random answer level and the accuracy in the complex condition, and anecdotal evidence for the opposite hypothesis. However, the ANOVA analysis did not provide evidence for a difference between the medium and complex conditions. Thus, we cannot conclude that the complex condition represents a clear limit for unconscious perception. Nevertheless, the complex decisions appear to be closer to such a limit than the simple decisions.

The results from the objective test of awareness provided moderate evidence that participants’ responses were random, which can be interpreted as evidence that participants were not aware of masked words and that the stimuli were indeed processed unconsciously. These results may have important implications for the discussion of the function of consciousness and may provide support for certain theories of consciousness.

### Function of consciousness

The present results do not allow us to identify a sharp boundary of unconscious processing. They suggest that masked stimuli can influence decisions not only when their semantic meaning is used directly, as in the simple condition, but also when this information has to be integrated with consciously presented contextual information, as in the medium condition. However, this influence was no longer reliably detectable in the complex condition, in which the use of masked information required an additional reasoning. Thus, although the complex condition may represent a candidate boundary condition in the present paradigm, the data do not allow us to conclude that the boundary begins precisely at the complex level of decision complexity.

A cautious interpretation is therefore that unconscious influence extends beyond the simplest semantic decisions but becomes increasingly unreliable as decisions require more complex integration or inferential processing. This pattern is consistent with the hypothesis that one of the functional roles of consciousness is to support the flexible use of information in higher-order cognitive processes.

It is worth noting that this does not imply that integration of information or reasoning are the defining features or evolutionary origin of consciousness. First, our study does not establish a precise boundary at which consciousness of masked words becomes necessary. Even if the complex condition is treated as a candidate boundary condition, or if the boundary lies somewhere between the medium and complex conditions, this would not imply that consciousness evolved late in evolutionary history, in animals capable of reasoning. This interpretation does not hold if we consider that, in this and in most other unconscious perception studies, the effectiveness of unconscious perception is extremely limited compared to conscious perception. For example, 58% correct responses in the simple condition still reflect near-random performance, whereas in catch trials accuracy approached ceiling levels. Thus, the present findings suggest only that conscious perception supports more effective reactions and decisions even in simple tasks, and may become increasingly important for more complex tasks, potentially including integration and inferential processing.

Moreover, even if integration of information or reasoning were to represent a boundary condition for unconscious processing, such a boundary should still be understood as reflecting only one of several functions that consciousness serves in the human cognitive repertoire. Simpler animals, which, for example, do not engage in logical inference, may nonetheless possess more basic forms of consciousness that fulfil different adaptive roles. These may include guiding attention to salient stimuli, supporting affective responses such as fear or pleasure, regulating motivational states, or enabling flexible behavior in response to changing environmental demands. In this context, the presence of consciousness should not be equated solely with the ability to reason, but instead understood as a graded, multifaceted phenomenon that can manifest in various forms across species and levels of cognitive complexity.

The results of the Decision Task may also be interpreted in the context of theories of consciousness. For example, according to GNWT, unconscious perception occurs in specialized networks that handle simple, automatic tasks, whereas more complex tasks requiring integration, contextualization, and reasoning are proposed to rely more strongly on globally available representations, which, according to the theory, are equivalent to conscious access (Dehaene and Naccache 2001) or conscious experience (Mashour et al. 2020). The observed decrease in performance beyond the simple condition is broadly consistent with this idea: unconscious perception of the masked stimulus (the masked premise) may suffice for simple decisions, whereas more demanding operations appear to place increasingly stronger constraints on the effective use of unconsciously perceived information. Thus, conscious perception of the stimulus, understood as its global availability for further processing (Mashour et al. 2020), may be necessary for cognitive operations that require integrating, maintaining, and using stimulus-related information across multiple processing systems.

However, the observation of above-chance performance in the medium condition requires further consideration. GNWT links consciousness of the stimulus to the global availability of information for flexible integration, report, and reasoning. The presence of an effect in this condition therefore suggests that at least some forms of contextual integration can occur without full global broadcasting (subliminal processing; Dehaene et al. 2006), whereas more demanding inferential processing—as in the complex condition—may place stronger demands on globally available representations (see also: Scott et al. 2018). Importantly, this observation does not constitute a direct test of GNWT. Rather, it indicates that integration of stimulus meaning with contextual information may represent a transitional level at which some effects of unconscious perception remain possible but become increasingly limited.

The absence of reliable priming in the complex condition is also consistent with accounts linking conscious perception of the stimulus to the global availability of information for flexible reasoning. Unlike the simple and medium tasks, the complex decisions required maintaining stimulus-related information over time, comparing it with stored knowledge, and applying an inferential rule. Such operations plausibly depend on interactions across multiple cognitive systems, including working memory, semantic processing, and executive control. In frameworks such as GNWT, these forms of multi-step, cross-domain processing are precisely those thought to benefit from globally available representations. The lack of robust priming in the complex condition is therefore consistent with the idea that unconscious perception may support simpler or more locally constrained operations, whereas inferential reasoning places stronger demands on globally accessible information.

The present findings may also be interpreted in relation to other theoretical frameworks of consciousness. For example, within recurrent processing theory (Lamme 2006), the results could suggest that the feedforward processing thought to support unconscious perception may be sufficient for relatively simple decisions but insufficient for more complex operations requiring integration and inference. Similarly, higher-order theories would interpret the pattern as indicating that first-order representations may guide simple behavior without conscious access, whereas integration or reasoning may require representations that are available at the higher-order level (Lau and Rosenthal 2011).

The current study expands the debate on the limits of unconscious perception and the extent to which higher-order processes can occur without conscious awareness. The existing body of evidence is mixed (Hirschhorn et al. 2021), with some studies challenging claims that unconscious perception enables high-level information processing (Kunde et al. 2003, Gelbard-Sagiv et al. 2016, Skora et al. 2023, Zher-Wen and Yu 2023), while others suggest that a limited degree of information integration may occur without awareness (Mudrik et al. 2011, Fahrenfort et al. 2017, Jiménez-Ortega et al. 2021, Zhao et al. 2021). Our study supports the idea that integration is possible to a limited extent; however, as in previous research, the evidence is not particularly strong. It seems reasonable to conclude that information integration—and especially integration combined with inference—lies very close to the limits of unconscious perception, or may constitute such a limit itself. This stands in clear opposition to the Unconscious Thought Theory (UTT), according to which deliberation can be effective, or even more effective, when it occurs unconsciously (Dijksterhuis 2004). In this sense, the present results add to existing critical arguments against UTT (Srinivasan et al. 2013).

### Limitations of the study

As described in the Experimental Procedure section, there is a qualitative difference between the simple condition and the two remaining conditions—medium and complex. In the medium and complex conditions, we used a consistent stimulus pattern that included both the Context Story and the Supraliminal Premise at the beginning of each trial. In contrast, the simple condition followed a slightly different pattern: the Context Story and Supraliminal Premise were replaced with a prompt to press a button and the Main Sentence Preview (this difference is clearly visible in Fig. 1). Consequently, participants in the simple condition received less information than in the medium and complex conditions, which may have influenced the results.

One possible approach to this problem would be to add some random, unrelated to decision information at the beginning of each trial in the simple condition instead of the button prompt. However, this might introduce a different issue: participants would encounter irrelevant information in the simple condition, while in the medium and complex conditions, the information would be relevant. An alternative might be to use both versions of the simple condition. Future research is necessary to explore this approach further and to clarify the potential impact of the type of information received on task performance.

The simple condition differs from the medium and complex conditions in another important aspect. Both the medium and complex conditions not only share a similar pattern of stimuli but also use the same content. For example, the “Mary and the physicist” stimulus appears in both the medium and complex conditions but not in the simple condition. We were unable to resolve this issue because the Subliminal Premise in the simple condition had to be positive or negative (e.g. “stupid” or “clever”), while the Subliminal Premises in the two remaining conditions had to be neutral (e.g. “a physicist” or “an accountant”). Nevertheless, it would be interesting to see an experiment with a design that addresses this issue.

Another limitation of the study is that our evidence for the null hypothesis (H0) in the complex condition (indicating no difference between random-level responses and participant scores) is anecdotal. Our interpretation of the results relies on the fact that we definitely found no evidence supporting the alternative hypothesis (which would indicate a difference between random-level responses and participant scores in the complex condition; H1). This lack of support for H1 is, in itself, valuable information. A replication with a larger sample size might help to strengthen this conclusion.

A potential weakness of the Decision Task is also that some context stories and sentences used in the study could suggest correct or incorrect answers, regardless of the subliminal stimuli they contain. For example, some names that we used in sentences could have had negative connotations and, as a result, influenced the choices of participants. Recall again the “Mary and the physicist” stimulus described in the Medium Condition section. If Mary wants to hire a physicist (medium condition) and the sentence is not “John is a physicist” but “Albert is a physicist,” it is possible that participants would answer YES to the question of whether Mary should hire Albert, even if they did not see the subliminal word. The reason is that the name Albert may suggest being a physicist because of the fame of Albert Einstein as a genius physicist. We dealt with this problem in various ways. First, during creation of stimuli, we chose as neutral names as possible. Furthermore, all sentences had two versions that counterbalanced the bias (when the word “physicist” was displayed subliminally, the bias would be toward the correct answer, but when the word “accountant” was displayed subliminally, the bias would be toward the incorrect answer).

Another potential bias could arise from the possibility that the Subliminal Premises in the medium and complex conditions might have had positive or negative valence (as in the simple condition) and could have influenced participants to give correct or incorrect answers regardless of the context stories. For instance, a physicist may be more positive to some participants than an accountant (or otherwise). This problem was solved analogously to the previous one: all the stories from the medium and the complex conditions had two versions. In one version, Mary wants to hire a physicist, while in the other, she wants to hire an accountant. Even if the word “physicist” might have more positive connotations than the word “accountant,” the bias was counterbalanced (in this case, the bias leads to the correct answer when Mary looks for a physicist but to the incorrect answer when she looks for an accountant). As mentioned in the Stimuli section, only one version of each stimulus appeared to a single participant.

Finally, this study has limitations concerning the measures of awareness. First, we did not include a subjective trial-by-trial test but rather used an objective measure. This approach was chosen because participants were already aware that subliminal stimuli were being presented—they had been asked to discriminate them during the initial calibration phase. Moreover, some words were presented supraliminally at the end of sentences in catch trials, and the overall task design clearly indicated that subliminal words appear in that position. Since all participants expected the presence of subliminal words, asking them whether they had seen anything could have elicited biased responses. Specifically, such questions could have produced false positives—claims of having seen something merely due to knowledge that something was supposed to be there. We also aimed to avoid extending the study, which was already lengthy and, in some cases, significantly prolonged by the calibration phase (as better discriminators required longer calibration). Future studies using this type of paradigm might address this issue by splitting the experiment into two sessions or including a long break mid-session to allow for the inclusion of additional awareness measures.

Relatedly, we did not include a subjective trial-by-trial awareness measure within the main task. In addition to concerns about false positives, embedding trial-by-trial questions into the main task posed additional challenges: the task was cognitively demanding. Participants had to process substantial information during each trial and maintain focus throughout the session, and adding an awareness question at the end of each trial could have further impaired performance (Biafora and Schmidt 2022).

Although not included in the main interpretation of our data, we conducted an informal debriefing session at the end of the study to gather additional subjective information about participants’ awareness. During this session, participants were asked a series of questions: Were the sentence endings words or pseudowords? Apart from the sequences of capital letters (masks), did you see any meaningful words at the end of the sentences? If so, approximately how many times did that happen? Were you able to read any of these words? If yes, how many times? If you managed to read any words among the strings of letters, please try to write them down.

This debriefing session was not intended as a primary measure of awareness. Rather, it served as an additional source of information to assess the extent to which participants consciously extracted information from the task and how many words they could recall. We were also interested in identifying which words—if any—might have been consciously perceived, to better inform future experimental designs (e.g. participants may have seen negative words in the simple condition).

A limitation of this informal debriefing is that participants were informed that all subliminal stimuli were either words or pseudowords, while in reality, they were all words. This instruction was intended to counterbalance potential bias stemming from participants’ knowledge of the study’s structure. However, it may have introduced an opposing bias, potentially exacerbating the criterion problem and reducing the reliability of the debriefing responses.

Nevertheless, the results of the debriefing session were generally consistent with the objective test of awareness. Three participants claimed they were able to read words presented between the masks, reporting an average of five meaningful words. However, only one provided a written response, listing two words—neither of which had actually been presented during the procedure. While these findings might suggest successful masking, we refrain from incorporating them into the main interpretation of the data due to the concerns outlined above.

## Conclusions

In conclusion, our results suggest that unconscious perception is highly limited, even in very simple decisions. Furthermore, the ability to use unconsciously perceived information in decision-making appears to decrease as decisions require more demanding integration and inferential processing. These findings are consistent with the idea that one of the functional roles of consciousness may be to support the flexible use of information in higher-order cognitive tasks.

## Data Availability

Data for this study are available at: https://osf.io/4dnj9/?view_only=6cb90f33adf04a9b882ea9933339e9c0.

## Footnotes

1 Alternatively, experiments that failed to find effects could be interpreted as evidence against the existence of unconscious perception. Numerous critiques have been raised regarding the methodologies used in unconscious perception research, suggesting that some reported effects may be artifacts stemming from methodological flaws (see, e.g. Kouider and Dupoux 2004, Shanks 2017, Meyen et al. 2022).

2 Sixty trials were intended; however, due to an error in the script procedure, 53 trials were presented to 21 participants, while the remaining participants completed 60 trials. The order of trials was randomized, so no specific trials were systematically excluded due to this error.

## Preprint

This work is being released as a preprint on OSF Preprints (doi:10.31234/osf.io/wvumr_v2), where it is publicly accessible.
